# Living through uncertainty: a qualitative study on leadership and resilience in primary healthcare during COVID-19

**DOI:** 10.1186/s12913-023-09223-y

**Published:** 2023-03-09

**Authors:** Soila Karreinen, Henna Paananen, Laura Kihlström, Kristiina Janhonen, Moona Huhtakangas, Marjaana Viita-aho, Liina-Kaisa Tynkkynen

**Affiliations:** 1 Faculty of Social Sciences, Tampere University, Tampere, Finland; 2 Welfare State Research and Reform, Finnish Institute for Health and Welfare, Helsinki, Finland; 3 Cultural, Behavioral & Media Insights Centre, Finnish Institute for Health and Welfare, Helsinki, Finland

**Keywords:** Resilience, Primary care, Primary healthcare, Leadership, Complexity, COVID-19, Finland

## Abstract

**Background:**

Resilience is often referred to when assessing the ability of health systems to maintain their functions during unexpected events. Primary healthcare forms the basis for the health system and thus its resilient responses are vital for the outcomes of the whole system. Understanding how primary healthcare organisations are able to build resilience before, during, and after unexpected or sudden shocks, is key to public health preparedness. This study aims to identify how leaders responsible for local health systems interpreted changes in their operational environment during the first year of COVID-19, and to elucidate how these views reflect aspects of resilience in healthcare.

**Methods:**

The data consist of 14 semi-structured individual interviews with leaders of local health systems in Finland representing primary healthcare. The participants were recruited from four regions. An abductive thematic analysis was used to identify entities from the viewpoints of the purpose, resources, and processes of resilience in the healthcare organisation.

**Results:**

Results were summarised as six themes, which suggest that embracing uncertainty is viewed by the interviewees a basis for primary healthcare functioning. Leading towards adaptability was regarded a distinct leadership task enabling the organisation to modify its functions according to demands of the changing operational environment. Workforce, knowledge and sensemaking, as well as collaboration represented what the leaders viewed as the means for achieving adaptability. The ability to adapt functioned to comprehensively meet the population’s service needs built on a holistic approach.

**Conclusions:**

The results showed how the leaders who participated in this study adapted their work during changes brought on by the pandemic, and what they viewed as critical for maintaining organisational resilience. The leaders considered embracing uncertainty as a principal feature of their work rather than viewing uncertainty as aberrant and something to avoid. These notions, along with what the leaders considered as critical means for building resilience and adaptability should be addressed and elaborated in future research. Research on resilience and leadership should be conducted more in the complex context of primary healthcare, where cumulative stresses are encountered and processed continuously.

## Background

### Resilience in healthcare

In assessing how healthcare can maintain its essential functions in a crisis, the concept of resilience is often raised [1]. Several frameworks conceptualising resilience have been suggested but a consistent definition for resilience has proved difficult to attain [2–6]. This may be because of the complex nature of both the phenomenon itself as well as the context of healthcare [7]. There are also several overlapping concepts that have been used to refer to when resilience is discussed in the context of health systems and healthcare [2, 5]. Concepts such as the health system or healthcare resilience address the topic from a broad system-wide perspective, while resilience in healthcare describes the phenomenon within the boundaries of an organisation or otherwise more restricted part of the health system. In this paper, we define, conceptualise, and analyse resilience according to Wiig et al. as “the capacity to adapt to challenges and changes at different system levels, to maintain high quality care” [8].

Crises affecting health systems have inspired an abundance of studies on resilience, especially after the Ebola virus or COVID-19 outbreaks [4, 9]. In a conceptualisation of organisational resilience Duchek [10] depicts the various dynamic connections between different resilience capabilities during the phases of unexpected events. According to this definition, resilience is a meta-capability consisting of a set of organisational capabilities and routines that allow for the successful accomplishment of the three resilience stages (anticipation, coping, and adaptation), which cannot be clearly separated. Elsewhere, researchers have argued that the study of resilience should take a broader view to account for both shocks and cumulative stresses to appreciate its complexity and context dependency [11]. In the field of quality improvement research, resilience is considered to be needed for longer periods and in the daily operation of healthcare units. Often referred to as everyday resilience, this approach is a rising research area [7, 12].

While a considerable amount of literature on resilience in healthcare is published at the system or hospital level, research in the context of primary healthcare is less commonplace [13] (see however, [14–19]). This is an omission, given that primary healthcare forms an important building block for strong national health systems [20] and most national health policies are brought to life in primary healthcare. Many of the core services of health systems are provided through primary healthcare such as health promotion and treatment for acute and chronic illnesses. Primary healthcare is also community-based care and often provides the first contact point in the whole healthcare system [21]. As a first point of contact, primary healthcare often engages with a substantial portion of the consequences of unexpected events. For instance, in the case of COVID-19, diagnostics, contact-tracing, and vaccinations have been a primary healthcare responsibility in many countries [22, 23]. Finally, service needs in primary healthcare are less defined and more outstretched than in specialised care [24] forming reasonable complexity in the operational environment.

Healthcare is commonly argued to be a complex adaptive system (CAS) [25, 26]. Typical characteristics of complex systems such as context specificity, ambiguousness, and their holistic nature set specific requirements for leadership [27–29]. Adopting a CAS approach emphasises that leadership needs to see and shape the system as a whole, that is, as a combination of different interacting and intertwined parts [26, 28]. This is contrary to traditional managerial thinking that considers organisations as a set of roles and easily defined parallel processes and solely as objects of management actions [30]. Due to its fundamental role for the whole healthcare system and the complexity it entails, it is important to understand better how crises are governed and managed in primary healthcare, i.e., what leadership entails in a complex adaptive system such as primary healthcare. This emphasises the need for health system leaders and scholars to understand a range of complexity issues, such as non-linear interactions and emergent behaviour and phenomena that affect leadership in primary healthcare organisations especially in uncertain circumstances [31–33].

Leadership and management are essential components and facilitators of resilience in healthcare [1, 5, 6, 13, 34]. According to previous research, resilient organisations are not managed hierarchically [10]. Instead, they employ decentralisation, self-organisation, and shared decision-making to allow for flexibility and adaptation [10], which are typical manifestations of CAS. Leadership focussing on complexity has been raised as one approach to nurturing resilience in healthcare [7, 28]. Khan Y. et al. [35] state that adopting a complexity lens can support leaders in managing change and transformation, which in turn, are fundamental elements of resilience. In summary, leadership approaches discussed in the resilience literature concerning healthcare usually include elements of participation, engagement, and collaboration. The connection between leadership and organisational resilience requires more empirical research and this would also be relevant to the healthcare sector [36, 37].

Against the presented background, this study contributes to the growing area of research on resilience in healthcare by studying how resilience in local health systems, including primary healthcare, was facilitated by leadership during the first year of the COVID-19 pandemic in Finland. More specifically, in this study we ask how leaders responsible for local health systems interpreted changes in their operational environment during the first year of COVID-19, and the role that leaders themselves viewed as important in building organisational resilience during a prolonged health crisis. Drawing upon interview data, we focus on leaders’ perceptions of local, primary healthcare level resilience and on the role of leadership in building resilience to maintain services in answer to population needs during an unexpected health system shock.

## Methods

### Setting and data collection

The context of the study is the Finnish healthcare system, which is a decentralised, universal health system. At the time of the interviews, primary healthcare was organised by municipalities. At the regional level, 20 hospital districts were responsible for organising specialised healthcare. In some areas, joint municipal authorities were responsible for organising both primary level healthcare and social services as well as specialised health services for the region [38, 39]. Municipalities have had a large amount of autonomy to decide on how they provide the services, but primary healthcare has been mostly organised through municipal health centres, which utilise a broad variety of professionals and services, including many public health functions such as the detection and treatment of communicable diseases, and vaccinations. Additionally, diverse chronic and acute illnesses are treated in primary healthcare instead of hospital settings. In Finland the integration of healthcare and social services has been one of the leading policy tools to develop the system [38, 40]. As of January 2023, a large-scale health and social services reform has taken place in Finland reducing the number of organisers of these services from over 300 municipalities to 22 wellbeing services counties. As a result of the reform, the responsibility for financing has recently been transferred to the state level [41].

To acknowledge the broadness of functions in the Finnish primary healthcare system, we use the term primary healthcare instead of primary care, the latter being usually understood as consisting of medical care for individuals provided by a family doctor or a general practitioner [42]. During the COVID-19 pandemic, municipalities in collaboration with primary healthcare were responsible for many public health activities, such as public communication, monitoring of case numbers, mitigation measures, quarantines, and isolation, as well as individual health needs (e.g., diagnosis, patient information, treatment of mild cases, and vaccination) providing the essence of the local health system response. During epidemics, hospital districts and the Finnish Institute for Health and Welfare (THL) support municipalities with their medical expertise [43].

The analysed data of this qualitative study were derived from 14 semi-structured interviews which were chosen from a larger data set (53 interviews) of a study focusing on resilience in the Finnish health system [44]. The interviewees of the larger data set represented health system actors at different levels of the health system. From that data set we were able to choose those informants who had a leadership role in local health systems and/or responsibility for leading the pandemic response in their organisation. The study participants were recruited from four regions in Finland, which differ based on their geography, demographics, and level of integration in health and social services, as well as their epidemiological situation during the point of data collection. At the time of the data gathering, the roll-out of vaccinations was only just beginning to accelerate. The regions are described in more detail in Table 1. The organisations are described according to the situation at the time of the study in 2021. One person asked to be interviewed for this subsample did not respond to the invitation, while all others agreed to participate. In order to ensure the confidentiality of the respondents, we have not combined information on the interviewees’ positions or other descriptions with the region they are representing. In a small country such as Finland the connection between a person and information given in the publication could be readily made.

**Table 1:** Description of the sampled regions in the study, including the number of participants

	**Organisation of health and social care system**^**a**^	**Population**	**Number of study participants**
Region 1	Joint municipal authority with eight municipalities, responsible for organising specialised as well as primary healthcare and social services in the region. One of the municipalities was not part of the joint municipal authority and had outsourced the social and primary healthcare services.	Low population density with approximately 70,000 inhabitants, approximately half of whom are centred around one municipality. The service needs are high due to the ageing population.	3 study participants
Region 2	Joint municipal authority with ten municipalities, responsible for organising specialised healthcare and most of the primary healthcare and social services in the region. For three municipalities, the primary services were outsourced to a private provider. Two of the municipalities in the region were not part of the joint municipal authority. The other had outsourced primary social and healthcare services to a private provider and the other arranged the services independently.	The region has approximately 200,000 inhabitants, while over half of them are centred to one municipality.	3 study participants
Region 3	A total of 23 municipalities in the region. The municipalities arranged primary healthcare and social services independently or through collaboration between municipalities. The hospital district was responsible for organising specialised healthcare.	The area has approximately 520,000 inhabitants, while almost half of them are centred in one city. The area has both urban densely populated areas and rural locations.	3 study participants
Region 4	Hospital district organised specialised healthcare in the region. Within the region, there were various arrangements for healthcare and social services.	The region has been the epidemic centre in Finland and has most experience in tackling a widespread epidemic. The region has approximately 1.7 million inhabitants, most of whom live in urban areas.	5 study participants

^a^The organisation of services are described as they were at the time of the study, prior to the health and social services reform which has taken place in January 2023

The 14 interviewees represent middle and upper-level leaders responsible for local healthcare and social services, including primary healthcare. The responsibilities and positions of the informants varied depending on the area in question. We also included city managers among the interviewees since the municipalities played a key role in pandemic governance in the Finnish context. The positions of the participants included city managers (*n* = 3), directors of joint municipal health care and social service authorities (*n* = 2), heads of social and health services in municipalities (*n* = 5), directors of health services (*n* = 3), and an administrative head nurse (*n* = 1). Due to the variety of positions and professional backgrounds of the participants, we use the term ‘leaders responsible for local health systems’. Primary healthcare is understood as part of the local health system. The term corresponds with the overall roles of the interviewees as they all are members of high-level boards or groups which are responsible for primary healthcare organisation and for the responses to the crisis. Purposive sampling was used to recruit key persons responsible for pandemic governance in each region. Potential interviewees were identified from public resources (e.g., media, organisations’ websites) and asking previously interviewed participants to name people in positions described above in their region. The research adhered with the ethical guidelines for responsible research completed with human subjects, and the participants provided verbal informed consent at the beginning of the interview. No compensation was offered to the participants.

The study tool used in the interviews was a flexible interview guide which built upon the shock cycle framework [1]. Accordingly, the interview guide included three main themes: preparedness for the pandemic, leadership and decision-making, and health system resilience including the themes of learning and adaptation. The interviews were conducted in March-June 2021 via Microsoft Teams and they were audio recorded and transcribed. Each interview lasted from 60 to 90 min and at least two researchers were present to ensure successful recording and other technical issues. The quotations from the interviews included in this study have been translated from Finnish to English by the research team.

### Analysis

In this qualitative study, we used an abductive thematic analysis approach to reach an in-depth understanding of the leaders’ perceptions and interpretation on resilience in healthcare [45]. Whereas the coding was conducted utilising a former framework [8], the formation of themes was as inductive as possible, bearing in mind that a researcher always relates one’s thinking to previous knowledge. The analysis consisted of iterative rounds of familiarisation with the data and coding with ATLAS.ti version 22. The coding and theme generation were both conducted by the first author. During the analysis and coding phase, the first author met regularly with the research team to receive feedback and critical commentary on the coding scheme.

To identify primary health care leaders' experiences regarding resilience in healthcare, our coding was guided by the four core questions suggested by Wiig et al. [8]. These investigate the different aspects of resilience in healthcare and are resilience *‘for what’, ‘to what’, ‘of what’*, and *‘through what’*. This four questions approach was chosen as it helps to focus on the processes and meanings of building resilience during crises and challenges. The questions also allow a broad consideration of resilience components on different system levels and contexts.

Against this background, the coding scheme was guided by the following questions and domains. The question concerning ‘*resilience for what’* elaborates on the purpose of resilience and asks: What goals and objectives does resilience support? The second question addressing *‘resilience to what*’ seeks to identify what is needed for resilience to become activated and asks: What are the triggers of resilience? The third question covering the *‘resilience of what*’, zooms in on which resources, material or immaterial components or participants that are used to build resilience in healthcare, while the last question concerning *‘resilience through what*’ uncovers what actions are taken to build resilience. In our analysis, we concentrate on two of the core questions: the resilience *‘of what’* and *‘through what’*. We consider the COVID-19 pandemic as a self-evident trigger of resilience and thus the core question of *‘resilience to what’* is less discussed in this paper although two triggers embedded in the pandemic situation were identified. As the aspects *‘resilience of what’* and *‘through what’*, include descriptions of resources and actions used to safeguard important services, they also indirectly illuminate the purpose of resilience (*‘resilience for what’*), the mentions of which were as such less frequent. We also included a fifth category in our analysis to address the deficiencies of resilience. This code group was named *‘insufficient or absent components of resilience’* allowing for reporting the leaders’ ideas on resilience in healthcare as a whole, acknowledging those aspects of resilience that were lacking or insufficient in their local health system context. In other words, the interviewees described several elements that they suggested were important elements of the healthcare organisation’s resilience, but which were not at hand or which they were unable to use.

The two aspects (resources or *‘resilience of what’* and actions or ‘*resilience through what’*) represented the vast majority (40 out of 45) of codes as well as quotations in the data. This can be partly explained by the questions presented to the interviewees, which concerned processes and resources used during the pandemic, as well as by the acuteness of the crisis which was still going on at the time of the interviews. At times, drawing a line between the question of *‘resilience through what’* and *‘resilience of what’* was challenging. For example, this was the case when communication or information flows were discussed, because information as a resource is so closely connected to the actions taken for effective information flows or public communication. Often an excerpt of text included both, and even insufficient or absent components of resilience. A systematic classification was followed to differentiate between processes (doing something) and resources (using something). In some cases, multiple codes were added to the same quotation. For example, the interviewees described that some information such as epidemiological indicators were readily collected and shared (*‘resilience of what’),* but knowledge about social care demands was more difficult to attain (*‘insufficient or absent components of resilience’*). When possible, a quotation from the interviews was divided into smaller sections and grouped into corresponding categories. If division was not possible without losing the association with the context, the same section was coded into all categories which it represented. The co-authors, who were also profoundly familiar with the data, commented on the formation and grouping of codes throughout the process in order to build a coherent code book.

**Fig. 1:**
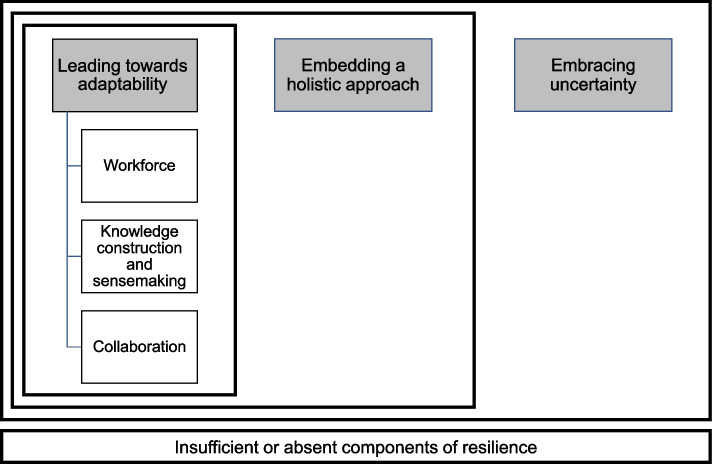
Themes and their hierarchical connections

The first author formed, assessed, and further developed the themes in close collaboration with the co-authors. A total of 45 codes were grouped under each core question addressing the aspects of resilience. The code groups of resilience components ('*resilience of what*') and processes supporting the organisations’ resilience ('*resilience through what'*) had the most mentions in the data and these groups also included the majority of the codes (22 and 18 respectively). The six themes identified as conceptual entities from the codes were organised hierarchically as they represent different levels and components of leadership and resilience. One of the main themes, leading towards adaptability, has three individual sub themes while the other two form more comprehensive entities themselves. The connections between the themes are illustrated in Fig. 1 and explained in the next section in a “bottom-up” manner.

## Results

In the results section, we present especially illustrative quotations from the interview data as examples of each theme. While the quotations selected for each theme represent a single interview, they have been selected based on their ability to illustrate aspects of the theme as a whole. Taken together, the themes represent what leaders responsible for local health systems viewed as essential to building resilience during COVID-19 (*‘resilience of what’*) and what actions they took (*‘resilience through what’*) [8].

### Leading towards adaptability

The study participants predominantly identified organisational adaptation as one of their major leadership tasks. According to the interviewees, the hierarchically governed Finnish public sector and using familiar modes of organising work were argued to be good grounds for building an adjustable crisis governance system. The constituents of early-stage adaptability also included using habitual modes of operation and organisation as well as the availability of material resources, although a lack of personal protective equipment for instance, was reported. Many organisations utilised the same management teams as usual to be able to make quick decisions, although these groups were soon complemented by new members and meetings were held more frequently.

In practice, adaptability meant changing modes of service or managerial activities to meet the new requirements in the operational environment caused by the COVID-19 pandemic. These modifications included, for example, meeting agendas, information gathering, as well as shifting resources back and forth between contact tracing, pandemic wards, and other tasks. These actions mirrored leaders’ needs to adjust operations for alternating demands in the course of the pandemic: the emphasis shifted between pandemic governance and giving the workforce (including the leaders) “some time to breathe and recover”. This was illustrated by one of the interviewees as follows:

“So, we shifted, we’ve been several times in a contingency plan mode and then again in non-contingency mode. So, we’ve had the principle that when we need an elevated alert level, we raise our alertness and as soon as the situation allows, we drop it again back to normal.” *(informant 6, director of joint municipal authority)*

Although the abovementioned example reflects the general need to adapt during the crisis mode of the first year of COVID-19 in Finland, we identified three sub-themes of adaptability. These were: leading the workforce, knowledge and sensemaking, and collaboration form the basis of primary healthcare organisations’ adaptability. These sub-themes of leading towards adaptability are addressed in the next sections.

#### Workforce

The leaders identified the healthcare workforce as a crucial resource and a key actor to achieve resilient responses. One way of ensuring an adequate workforce was the reallocation of staff, which was carried out also across sectoral boundaries at the municipal level. The reallocation generated the need to introduce new tasks and to ensure continuous support for the employees in the changing working environment. Mostly the experiences were positive, as described by one of the interviewees:

“But I have to say that from the viewpoint of the whole city, for example the staff from cultural services like libraries, museums, the city orchestra, and from other places, they have been ready to come and do really different kinds of jobs on a short notice when their operations were closed (due to COVID-19). It has in a sense opened the eyes of some in those sectors but also in social and healthcare services that we don’t have to limit ourselves solely to the workforce representing social and health care. That it is worth taking a broader perspective in crises, looking at the resources society has to offer.” *(informant 5, head of social and health services (municipality))*

The high morale and motivation of the workforce during the first months of the pandemic was praised, and some organisations even experienced reduced absenteeism. However, the leaders also questioned whether the support for the employees was sufficient. They acknowledged that the organisations had not prepared well enough for the strain on workforce, and that measures to support the employees were not included in the contingency plans of their organisations. All this was further emphasised as the pandemic and thus extraordinary circumstances were prolonged. One of the informants noted:

“But people get tired when the situation lasts for a long time, that’s for sure. It happens to the leaders, to the managers, and to everyone who is involved. We are trying to implement a COVID exit at this stage, so that people can have their vacations, and that they get feedback on their work, and all. It is a big question.”* (informant 4, director of joint municipal authority)*

In sum, the leaders acknowledged the importance of a motivated workforce for resilience in healthcare. Simultaneously, they considered that the personnel had not always been supported well enough and were concerned about the long-term effects of the pandemic and various other stresses on the workforce.

#### Knowledge construction and sensemaking

Collecting, combining, and analysing information from different sources are a cornerstone for resilience in healthcare. A common view amongst the interviewees was that the staff competencies and knowledge accumulated prior to the crisis were pivotal, especially at the beginning of the pandemic. Different preparedness activities, such as contingency planning, exercises, and simulations, which are organised regularly, were regarded helpful although examples of insufficient preparedness or experience were cited as well:

“Somehow at this moment I think even more, if we talk about preparedness planning, that working on different scenarios should have been done more, that before this situation sketching, outlining scenarios could have been made. Now we have been leaning on the experiences from previous pandemics a bit too much and it would probably have been useful to carry out more planning and preparations with other actors.” *(informant 5, head of social and health services (municipality))*

As stated by the interviewees, knowledge management including analysing information was seen as central in crisis governance and thus accessible, although often incomplete, information was described as a foundation for decision-making. One of the interviewees noted:

“It has been really successful in the sense that the situational awareness that we have at hand at any time, it’s been accessible as frequently as daily or several times a day. Each time we went through the situation and after that we could propose if something needed to be done or not. I don’t remember any occasion that something that was well argued hadn’t been carried out.”* (informant 7, director of health services (joint municipal authority))*

Collecting and combining information was also seen as important for learning from the previous phases of the pandemic and preparing for the next phase. Sharing information, analysing and sensemaking tasks were often carried out in different groups or networks which are discussed under the next theme.

#### Collaboration

New collaborative practices were introduced during the pandemic to enforce coordination and pandemic governance. These included the creation of new expert groups, but also complementing existing ones or adding to the frequency of regular meetings. Communication through these groups often functioned to combine stakeholder views and sensemaking. One of the interviewees explained this as follows:

“We’ve been aware of their (the local hospital district) situation at all times, and they’ve been aware of ours. Together, we resolved issues such as where to set up the infection wards and how the patient flows would be organised. This still goes on. We meet two times per week and keep each other on track.”* (informant 4, director of joint municipal authority)*

Trust and prior familiarity between stakeholders appeared as facilitators for collaboration during the pandemic. Representatives from organisations with integrated social and healthcare services estimated that pandemic governance was more effective since both sectors “sat around the same table” and were under the command of the same leader, which exemplifies one structure that promoted collaboration between sectors. The clarity of stakeholder roles and responsibilities was an important benefit, enabling timely and effective organisational responses. However, the interviewees expressed that this clarity was often missing, thus, undermining the ability to adapt quickly to the new situation.

In addition to collaboration between the sectors of the same organisation, collaborative groups and networks played an important role in pandemic governance on the regional level (i.e., collaboration between municipalities and between municipalities and other key organisations such as hospital districts). The foremost aim of inter-organisational collaboration was to find a consensus on decisions to be made throughout the region. One of the informants described the collaboration:

“First, between our people, and then with the municipal leaders and leaders of the education sector, we communicate about what we think of any issue at hand. Then, it may be that they think in one way and we in another, but we don’t go with that mindset to the meeting, we work out our opinion and then we want to hear what THL (Finnish institute for health and welfare) thinks. But the starting point in these COVID-coordination groups is that from THL’s point of view they too want to know what the region’s opinion is. And then it’s good that the region has an opinion. We can discuss that ”we have these viewpoints, but this is our solution.”* (informant 11, director of health services (joint municipal authority))*

In a large-scale health crisis such as COVID-19, municipalities and local health systems were naturally part of a larger system for pandemic governance. This increased the demand for leaders to participate in several networks. Different mentions on collaboration were notably frequent in the interview data underlining its significant role in the leaders’ work during the pandemic.

### Embedding a holistic approach

The leaders described their view of the crisis and population in many ways that emphasised their holistic approach to the situation. They underlined the need to have a comprehensive picture of the diverse population needs and the effects of the crisis on the population and services. The overall well-being of the population can be interpreted as the evident purpose of resilient responses and activities to maintain population trust. This includes adequate services and clear public communication, which were considered critical by the interviewees as explained here:

”Regardless of the situation, we discuss issues beyond healthcare. I think that an integrated healthcare and social service organisation like this is very fruitful, because in the same steering group we can always look at the situation from the viewpoint of child welfare to intensive care. It’s a broad perspective, and it enables us to discuss a wider array of issues than one would if you only look at it from the angle of healthcare.”* (informant 6, director of joint municipal authority)*

Balancing the multifaceted needs of the population and the realities of service production, such as financing and availability of workforce, were recurring themes in the interviews, often declared as insufficiently managed. Some services were scaled back during the first months of the pandemic to secure the capacity in other parts of the health system. However, the informants felt that these adaptations could not be prolonged as the pandemic went on and they also reprehended some other societal mitigation measures, such as school closures, as they held the potential to aggravate citizens’ overall wellbeing. As one of the informants reflected:

“I think this is a good lesson, an observation, for us that if everyone just does their business, then this thing will not work out. So, we need to recognise the pressures and situations that arise when the health authority decides or recommends something, that what kind of a chain starts from there and how that will be handled. For me the acknowledgement and understanding of these cascading impacts has been an essential observation. They are highlighted in this situation and it’s something that we all have probably learnt.”* (informant 13, city manager)*

This balancing is an important task for leaders, in order to ensure both sufficient adaptability and stability. Embedding the holistic approach is reflected in the three categories: the purpose of resilience ('*resilience for what'*), the immaterial resources of resilience (e.g., financing and understanding the multifaceted effects of the crisis), and processes of resilience (e.g., building a holistic view of the service needs) all aiming at supporting the wellbeing of the population.

### Embracing uncertainty

The final theme covers uncertainty as characteristic to the local level health services and leadership. The interviewees stated that the key persons should be able to make decisions and work effectively during uncertain times. This seemed to have succeeded well with very few exceptions. Every crisis or shock was acknowledged to be of a different kind and only partial preparation for the next one is possible since no one has the certainty as to what the next crisis will be. Multiple shocks and cumulative stresses, such as questions of workforce availability and the future health and social services reform taking place as of January 2023, were also mentioned. Functioning as an interpreter of uncertain situations is a principal endeavour for leadership, and the interviewees believed leaders to be more apt at this skill than front-line workers. One of the informants illustrated this as follows:

“It can be easier for senior level leaders to live under uncertainty and to put up with the world turning into 24/7 reality. But I think we should have thought more about the staff coping on that level. It doesn’t help much if you’re told that this is what it’s like now and let’s just work it out when there are so many uncertainties out there. When you’re in a leading position you possibly better tolerate the uncertainty and that you don’t know what tomorrow brings. But it’s so important for people to know about their days off and holidays and what they’re allowed to do."* (informant 10, head of social and health services (municipality))*

The undoubtedly significant role of instability was stressed by the leaders on many occasions, such as in relation to preparedness plans, the epidemiological situation, national instructions, and the sufficiency of workforce. However, the interviewees also listed several uncertainties that were present before the pandemic started and portrayed the constant change as a prevailing circumstance in their operational environment. The leaders would like the workforce to be able to adapt to continuous change. Hence, it can be interpreted that “living with uncertainty” instead of just tolerating it, is an important part of resilience in primary healthcare.

## Discussion

In this study we analysed the interpretations of the leaders at the local health system level on the changes in their operational environment during the COVID-19-pandemic and, furthermore, how these interpretations reflected aspects of resilience in healthcare. We detected that leading towards organisational adaptability, embedding a holistic approach in operations, and embracing uncertainty are essential aspects of building resilience in healthcare. Our results also highlighted the importance of considering absent or insufficient components of resilience in healthcare in operationalising the concept. Wiig et al. [8] have defined resilience in their study context as “the capacity to adapt to challenges and changes at different system levels, to maintain high quality care”. In the light of our data and analysis, resilience in primary healthcare expands to signify a capacity to adapt to unforeseen events and changes at different system and organisational levels, and in different sectors regardless of uncertainties, to maintain services to answer to population needs and to support the population’s overall wellbeing.

Local governance has been identified as an important part of resilience in healthcare and for the pandemic response [6, 46, 47]. The Finnish decentralised and publicly funded healthcare system serves as an example of the role of local governance in the context of the pandemic response, which can generally be regarded as one of the strengths of Finland’s pandemic response [43]. This paper contributes to the literature of resilience in healthcare by studying how resilience is interpreted by leaders at the local health system level, which, in turn, increases the understanding of how to strengthen health system resilience overall [48].

In the context of primary healthcare, the need for horizontal and vertical collaboration are accentuated especially during a crisis—our interviewees participated in several collaborative groups with stakeholders on different health system levels. Those participants who represented joint municipal authorities in our study worked in the type of organisations where different levels of healthcare and social services are integrated. These participants declared clearly to be in favour of an integrated structure as it facilitated collaboration between sectors and health system levels. The same structure of organisation has been formed in other Finnish regions as well as part of a major health and social services reform from January 2023 on. While these participants’ notions supported the integration of healthcare and social services in Finland, it is important to bear in mind that the benefits of local governance should not be lost when services are being centralised. Equal attention should be paid to ensure that primary healthcare is recognised as playing a key role in preventive functions and public health interventions [6].

Leadership is a critical building block of health systems and pivotal in supporting resilience in healthcare, yet it is rarely explicitly centred in resilience studies [6, 13]. Several frameworks portray resilience as a process or a collection of capacities or capabilities incorporating a considerable degree of dynamics and interdependencies [1, 10, 49]. According to our results, leaders responsible for local health systems played an active role in building resilience during the COVID-19 pandemic in Finland. Our analysis complements the notion that different components and aspects of resilience are not only strongly interdependent, but also entwined [10, 37]. The results support the findings by Lyng et al. [34] who articulate that resilience in healthcare requires a holistic awareness of a wide array of resilience capacities, which have to be maintained simultaneously, instead of concentrating on just a few of them. The presence of the future health and social services reform in Finland also caused considerable cumulative stress and uncertainty in the healthcare organisations and put pressure especially on the leaders.

The non-linearity of the chain of events triggered by the COVID-19 pandemic is another evident observation derived from the data. The results illustrate how steps were taken back and forth during the pandemic in healthcare organisations, and the crisis held unexpected events within itself, such as pandemic waves and challenges of healthcare workforce availability. The stages of the resilience process (anticipation, coping, and adaptation) could not be clearly separated in the data, as Duchek also presents in her conceptualisation [10].

As the present study also illustrated, the need to embrace uncertainty is pronounced during crises and in complex surroundings such as local health systems and primary healthcare [26, 50]. In order to nurture resilience in healthcare, the interviewees adopted a holistic approach and described attempting to incorporate uncertainty as an existential orientation of the system they were leading. They also used several means of promoting adaptability by leading the workforce, striving for sensemaking of the knowledge that was gathered, and collaborating in various networks. These endeavours reflect leadership focussing on complexity, also referred to as complexity leadership, in which the leader’s role is more about building connections for emergent adaptability, as well as sensing opportunities and challenges than trying to control the “messiness” of the complexity [26, 51].

Furthermore, our results illustrated how the interviewees reported insufficiency or absence of several aspects in their organisational environment that restricted or challenged their ability to respond to the crisis, such as scant resources, inability to support the workforce, insufficient information flows and the deficient quality of preparedness plans. We interpreted these as representations of missing parts of resilience in healthcare. Based on this finding, we argue that acknowledging the missing aspects of resilience is as crucial from the perspective of a comprehensive crisis response as an awareness of those aspects of resilience that are present. In this study we concluded a fifth category of *‘insufficient or absent components of resilience’* to our analysis in addition to the core questions introduced by Wiig et al. [8] answering the question ‘What does the healthcare organisation’s resilience lack?’. However, the insufficient or absent components of resilience in healthcare that we were able to identify were the same as those that the informants identified as present components. Thus, we suggest that the aspect of possibly absent or insufficient components of resilience in healthcare be actively considered in study designs of future research. It is noteworthy that our results represent only those insufficient components of resilience that were explicitly identified by the interviewees. Therefore, it is an important task for further studies to continue to investigate other potential shortcomings of crises responses that could be identified by using alternative data collection methods and research designs. The interviewee selection may also have affected the identification of lacking components. The resources or capabilities that have not been identified should be of high interest for both scholars and the leaders of health systems and healthcare organisations in the tasks of anticipation and learning and will likely require multimethodological approaches, including observational studies in healthcare settings [52, 53].

### Limitations

Our data consisted of 14 interviews which were utilised to conceptualise leaders’ experiences of resilience in healthcare in the context of local health systems and primary healthcare in Finland. Primary healthcare and public health crises, as well as leadership and resilience, are complex issues with an abundance of variance in different contexts. However, our study protocol addressed this pitfall by including interviewees from four different regions and, at least in the Finnish context, the findings were analogous.

Because of the relatively limited number of participants in the study, generalisations of findings must be made with caution. Nonetheless, the study provided a deeper understanding of leaders’ perceptions of resilience in a local health system setting, as well as conceptual considerations of resilience in healthcare, which can be used in further research. Furthermore, it is important to note that assessing the extent of the achieved resilience in healthcare is beyond the scope of this study, and therefore remains an important research task for further studies [54]. The themes of the semi-structured interviews naturally had an impact on the contents of our data. For example, questions on preparedness and workforce were asked which might have led the interviewees to contemplate these aspects more carefully and therefore also to identify the shortcomings in these sectors. A study protocol with a more open narrative inquiry could confirm or challenge the results of this study.

### Implications

The practical implications of this study arise from the finding that different aspects of resilience in healthcare, leadership roles, and stages of the crisis cannot be separated, nor lines strictly drawn between their components. Before, during, and after a crisis, leaders should think and act with a broad mindset, which can be facilitated by inviting other members of the organisation as well as other stakeholders to sensemaking discussions to build a comprehensive understanding of the operational environment and pressures directed to the organisation. Health workforce and their low turn-over rate, along with other resources that support resilience, are key in achieving agility of the organisation and ensuring the continuance of services for the population. Future research should ensure that resilience is explored with a broad view, also investigating whether same issues are of importance in leadership and managerial tasks in everyday resilience as in resilience triggered by exceptional shocks.

In Finland, the Ministry of Social Affairs and Health has provided guidance for operators in the healthcare and social welfare sectors for preparedness planning and continuity management in 2019 [55]. The guidance aimed at providing practical instructions for developing preparedness plans at the operational level. However, based on our results, although preparedness plans were made, they were not in all respects feasible for the COVID-19 pandemic. For instance, inter-organisational collaboration and scenario work played a much larger role during the pandemic than had been taken into consideration in the preparedness plans, and workforce issues had scarcely been addressed. Thus, there is an essential need for developing the national guidance for preparedness planning at operational level and providing practical instructions for primary healthcare leaders. For example, the results of this study emphasise the importance of planning how the workforce will be supported during crises, especially long-lasting ones.

## Conclusions

The findings of this study accentuate the importance of a holistic approach and ability to work under uncertain conditions at the local level of a health system and in primary healthcare in particular, especially during a crisis. This broad and multifaceted mindset seemed to be intrinsic to the study participants who were leaders responsible for local health systems in Finland. It is apparent that leaders are in a key position in building and maintaining resilience before, during, and after a crisis. Complexity leadership can provide beneficial approaches to both leaders and researchers in operationalising resilience in healthcare. Our findings confirm that the governance of unexpected events should accommodate different aspects, such as the workforce and other resources as well as processes that support resilience and stages of a crisis simultaneously. This entwinement should be emphasised in research and policy recommendations. Simplifying the complex nature of resilience in healthcare should be avoided.

## Data Availability

The datasets generated and analysed during the current study are not publicly available due to European provisions on data protection, of which the interviewees were assured.

## Abbreviations

CAS: Complex adaptive system
THL: Finnish institute for health and welfare
